# A New Scale for Predicting the Risk of In-hospital Mortality in Patients With Traumatic Spinal Cord Injury

**DOI:** 10.3389/fneur.2022.894273

**Published:** 2022-06-02

**Authors:** Yining Gong, Jinpeng Du, Dingjun Hao, Baorong He, Yang Cao, Xiangcheng Gao, Bo Zhang, Liang Yan

**Affiliations:** Department of Spine Surgery, Honghui Hospital, Xi'an Jiaotong University, Xi'an, China

**Keywords:** traumatic spinal cord injury (SCI), mortality, in-hospital, factors, scale, multivariate logistic regression analysis, epidemiological investigation

## Abstract

**Purpose:**

To analyze the relative factors influencing in-hospital mortality in patients with traumatic spinal cord injury (TSCI), and develop a score scale for predicting the risk of in-hospital mortality.

**Method:**

We reviewed the medical records from 59 spine centers in mainland China from 1 January 2018 to 31 December 2018. The inclusion criteria were (1) confirmed diagnosis of TSCI, (2) hospitalization within 7 days of injury, and (3) affecting neurological level from C1 to L1. The exclusion criteria were (1) readmission, and (2) incomplete data. Included patients were classified into the survival and non-survival groups according to their status at discharge. Univariate and multivariate logistic regressions were performed to identify the factors related to in-hospital mortality in patients with TSCI. A new scale was developed, and the mortality rate in each risk group was calculated.

**Results:**

Of the 3,176 participants, 23 (0.7%) died in the hospital, and most of them died from respiratory diseases (17/23, 73.9%). After univariate and multivariate logistic regression analysis, cervical spinal cord injury [odds ratio (OR) = 0.264, 95% confidence interval (CI): 0.076–0.917, *P* = 0.036], abdominal visceral injury (OR = 3.778, 95% CI: 1.038–13.755, *P* = 0.044), the American Spinal Injury Association (ASIA) score on admission (A: reference; B:OR = 0.326, 95% CI: 0.093–1.146, *P* = 0.081; C:OR = 0.070, 95% CI: 0.016–0.308, *P* < 0.001; D:OR = 0.069, 95% CI: 0.019–0.246, *P* < 0.001), and surgery (OR = 0.341, 95% CI: 0.146–0.796, *P* = 0.013) were significantly associated with in-hospital mortality. Scores for each of the four factors were derived according to mortality rates. The sum of the scores from all four factors was included in the scoring system and represented the risk of in-hospital mortality. The in-hospital mortality risk of the low-risk (0–3 points), moderate-risk (4–5 points), and high-risk groups (6–8 points) was 0.3, 2.7, and 9.7%, respectively (*P* < 0.001).

**Conclusions:**

Cervical spinal cord injury, abdominal visceral injury, ASIA score on admission, and surgery were significantly associated with in-hospital mortality in patients with TSCI and stable condition. The scale system may be beneficial for clinical decision-making and for communicating relevant information to patients and their families.

## Introduction

Traumatic spinal cord injury (TSCI) is defined as damage to the spinal cord with an external physical impact that temporarily or permanently causes changes in its function (1). The incidence of TSCI varies among countries and ranges from 8.0 per million persons to 49.1 per million persons, but it has not changed significantly over the recent years (2, 3). TSCI has devastating consequences for patients, their families, and society at large, due to the related loss of independence, high incidence of complications, and significant medical cost burden (3, 4). Timely diagnosis and implementation of neuroprotective interventions should be the first consideration in the treatment of TSCI to save progressively lost functional neural tissue. Key neuroprotective therapies involve surgical intervention, methylprednisolone administration, and thorough hemodynamics management (5).

Over the last three decades, considerable advances have been made in the management of patients with TSCI, both surgical and nonsurgical (6). However, the overall mortality rate in patients with TSCI is still ~2.8 times higher than that in the general population (7). The mortality rates of TSCI are very high in hospitals, and this in-hospital mortality rate gradually increased yearly from 6.6% in 1993–1996 to 7.5% in 2010–2012 in the United States (1, 8). However, the mortality rate decreases after hospital discharge, being 3.8% during the first year post-injury, 1.6% during the second year, and ~1.2% for the following 10 years (9). Advances in medical practice have led to changes in the causes of death in individuals with TSCI. Previously, renal failure and other related urinary tract complications were reported to be the primary causes of death in individuals with TSCI (10, 11). However, at present, the leading causes appear to be conditions commonly reported in the general population, such as cardiovascular diseases, infections, and respiratory diseases (12, 13).

Previous studies have reported several predictors of mortality in TSCI, such as age, neurological level, severity of neurological deficits, comorbidities, presence of other injuries, and ventilation status (7, 14–17). However, the sample sizes of most studies did not yield convincing findings, and their long time span involving changes in treatment may have affected the analysis. Besides, in Chinese health system, patients with TSCI and unstable condition are mostly treated in the emergency department, especially those with unstable vital signs. Only patient whose condition is stable or stable after being treated by the emergency department will be admitted to the wards of spine centers for further surgery or other treatments. However, we do not know the survival prognosis of this subset of patients. About 3,000 tertiary hospitals have set up orthopedic departments in China, some of which have specialized spine centers. Here, we aimed to analyze factors and designed a new scale system for predicting the risk of mortality in patients with TSCI and stable condition using data from multiple centers within a short period and with a focus on in-hospital stage.

## Materials and Methods

### Study Design and Participants

This cross-sectional study is a secondary data analysis of data from the national epidemiological investigation of TSCI in mainland China in 2018. The study was approved by the institutional review board of Honghui Hospital (No. 201904001), and the need for informed consent was waived due to the retrospective nature of the study and the absence of any intervention. We used a multistage stratified cluster sampling method to enroll a sample of spine centers which would be representative for this study. In stage 1, sample size calculation estimated a need for a total of 90,276,111 inhabitants in the whole country according to Poisson distribution (18). Then, at least 22 cities should be selected after calculated in equal proportions with the national population and the number of cities in 2018:


$$\begin{array}{l} \text{n }\left(\text{selected cities}\right)\text{=}\frac{\text{total sample size in the whole country }}{\text{national population in 2018}}\\ \;*\text{ number of cities in 2018}. \end{array}$$

In accordance with the criteria of the National Bureau of Statistics for the classification of the three major economic zones in the east, central, and west, the sample is divided into three layers, and the cities are finally expanded to 27, with nine cities in the east, central, and west, respectively. In stage 2, considering the siphon effect of competitive hospitals on provincial capital and the referral patterns adopted in TSCI, 3 provincial capital cities, 3 cities geographically adjacent to provincial capital, and 3 cities geographically not adjacent to provincial capital were selected with simple random sampling in each layer. In stage 3, 59 spine centers (18 centers in the east, 20 centers in the central, and 21 centers in the west, respectively) were finally included considering the qualification to treat patients with TSCI and the feasibility of the plan.

We reviewed the medical records of these spine centers from 1 January 2018 to 31 December 2018, and patients were selected using the International Classification of Diseases-−10th version—Clinical Modification (Supplementary Material 1) and the diagnostic code for TSCI. The inclusion criteria were as follows: (1) confirmed diagnosis of TSCI, (2) hospitalization within 7 days of injury, and (3) affecting neurological level from C1 to L1. The exclusion criteria were as follows: (1) readmission, and (2) incomplete data. To prevent oversampling bias, patients were only included in the first admission when they were referred to other hospitals. Patients were classified into the survival and non-survival groups according to their status at discharge.

### Data Recording Form

We designed a data recording form, according to relevant epidemiological research on TSCI globally, and advice from epidemiological and clinical experts at Xi'an Honghui Hospital. The final version of the data recording form (Supplementary Material 2) was created after being modified several times by experts and through a pilot test. The established database included the following items: (1) general information and demographic characteristics (age, sex, and occupation); (2) relevant information regarding the TSCI (date of injury, cause of injury, date of admission, injury level, American spinal injury association (ASIA) score, and spinal fracture), (3) combined injuries to other organs, (4) treatment (surgery or not, and surgical procedures), and (5) death and the cause of death. Generally, ASIA scores were evaluated for the examination and classification of sensorimotor impairments in patients on admission by resident doctors with orthopedic qualifications in the spine center, and checked by senior doctors, according to ISCNCSCI assessment (19, 20). The degree of neurological impairment was divided into 4 grades, that was A, B, C, and D, the impairment in the former was more serious than that in the latter.

### Data Collection

Spine centers that were qualified to treat patients with TSCI were selected by multistage stratified cluster sampling. We searched the medical records of each participating hospital and extracted the data of patients who met the criteria. The investigation team comprised graduate students and doctors who specialized in spine care. All investigators were trained before data collection, and a manual of procedures with detailed instructions for data recording form administration and data entry was provided. Two investigators were assigned to one unit during the investigation, and they separately extracted and cross-checked the data. Any problem was solved through timely discussion and consultation with experts. To ensure that data was accurate, double entry of the data recording form data was independently carried out by two staff members. After the database was established, the epidemiologist checked the data, and incomplete or abnormal values were corrected by checking the data recording form or original medical records. All corrections were confirmed by the corresponding investigators, epidemiologists, and supervisors.

### Statistical Analysis

Data were entered using Epidata version 3.1 (Epidata Association, Odense, Denmark), and Microsoft Office Excel 2019 (Microsoft Corporation, Redding, WA) was used to establish the database. All analyses were performed using SPSS version 26 (IBM, Armonk, NY). Frequency distributions and chi-square tests were used to describe and compare the mortality rates of each study population. Univariate logistic regression analyses were performed to examine the associations of age, sex, occupation, cause of injury, fractures or dislocations, cervical spinal cord injury, head injury, chest injury, abdominal visceral injury, time before hospitalization, ASIA score on admission, time before surgery, and surgery with the outcome of in-hospital mortality. Factors with *P* < 0.20 were entered into the multivariate logistic regression and results were reported as odds ratios (ORs), 95% confidence intervals (CIs), and *P-*values. Non-significant variables after multivariate logistic regression were excluded from the final regression model. Statistical significance was set at *P* < 0.05.

We designed a new scale system for predicting the risk of TSCI-related in-hospital mortality according to the risk factors derived from multivariate logistic regression analysis. The primary score of each item was determined by the in-hospital mortality rate. For convenience of clinical application, we simplified the scores mathematically. First, we subtracted the lowest value of each item from the value of the item itself, obtaining the adjusted scores. Then, each value was divided by 7 to get the nearest integer, leading to the final scores. The total scores were obtained by adding the individual scores and ranged from 0 to 8 points. Based on their scores, patients were divided into three groups. The low-risk group (0–3 points) contained either several 1-point items or only one 3-point item. The moderate-risk group (4–5 points) contained one 3-point item and one or two 1-point items. Finally, the high-risk group (6–8 points) contained two 3-point items with or without several 1-point items.

## Results

### Demographics and Characteristics

The study involved 59 tertiary hospitals of mainland China and a total of 3,176 patients (Eastern region: 1,269/3,176, 40.0%; Central region: 976/3,176, 30.7%; and Western region: 931/3,176, 29.3%). Of the total patients included in the sample, 2,409 (75.9%) were male and 767 (24.1%) were female. Age ranged from 1 to 92 years [mean (standard deviation), 51.0 (14.8) years]. The proportions of patients aged <60 years and ≥60 years were 69.5% (2,206/3,176) and 30.5% (970/3,176), respectively. Patients with TSCI were more likely to be farmers (1,331/3,176, 41.9%) and workers (424/3,176, 13.4%) than others. Traffic accidents (962/3,176, 30.3%), falls from height (905/3,176, 28.5%), and tumbles (865/3,176, 27.2%) were the three most common causes of injuries. The median time between injury and hospitalization was 14 h (ranged from 0 to 168 h). There were 1,070 (33.7%), 709 (22.3%), and 1,397 (44.0%) patients whose time between injury and hospitalization was <8 h, 8–24 h, and ≥24 h, respectively.

Regarding the level of injury in TSCI, 2,308 (72.7%) and 868 (27.3%) patients had cervical spinal cord injury and thoracolumbar spinal cord injury, respectively. ASIA scores on admission were recorded, and 30.3% (960/3,176) of the patients were classified as ASIA A or B. In total, 2,023 of 3,176 (63.7%) patients had spine fractures. A combined injury was observed in 51.6% (1,640/3,176) patients, and 1,019 (32.1%) patients had a head injury, 841 (26.5%) patients had a chest injury, and 104 (3.3%) patients had an abdominal visceral injury. After admission, 70.2% of patients (2,230/3,176) underwent surgical treatment. The median time between injury and surgery was 32 h (ranged from 3 to 188 h). Among 2,230 patients who underwent surgical treatment, 63.5% (1,417/2,230) patients underwent decompression and fixation fusion, 27.0% (603/2,230) patients underwent decompression fixation without fusion, 4.5% (100/2,230) patients underwent simple decompression, 4.9% (110/2,230) patients underwent other procedures. Besides, posterior and anterior approaches accounted for 60.6% (1,352/2,230) and 35.0% (780/2,230), respectively.

### In-hospital Mortality

Of the 3,176 participants, 23 (0.7%) died in the hospital. The median time from admission to death was 16 days (ranged from 1 to 108 days). Patients aged ≥60 years (10/970, 1.0%) had a slightly higher mortality rate than patients aged <60 years (13/2,206, 0.6%); however, there was no significant difference (*P* = 0.179). Male (16/2,409, 0.7%) and female (7/767, 0.9%) patients had similar mortality rates (*P* = 0.468). Regarding causes of injury, falls from height (10/905, 1.1%) and traffic accidents (9/962, 0.9%) caused higher mortality rates than others, and there was no death from sports injuries. Patients with cervical spinal cord injury (20/2,308, 0.9%) had a slightly higher in-hospital mortality rate than those with thoracolumbar cord injury (3/868, 0.3%), without statistical significance (*P* = 0.159). Patients with chest (11/841, 1.3% vs. 12/2,335, 0.5%, *P* = 0.030) or abdominal (3/104, 2.9% vs. 20/3,072, 0.7%, *P* = 0.038) injuries had higher in-hospital mortality rates than those without chest or abdominal injuries. The mortality rate gradually decreased as ASIA scores changed from A to D (*P* < 0.001). Patients undergoing surgery (11/2,230, 0.5%) had a lower mortality rate than those who did not undergo surgery (12/946, 1.3%) (*P* = 0.036) (Table 1).

**Table 1:** Mortality by demographics and characteristics.

**Characteristics**	**Total**	**No. of Patients died**	**Mortality (%)**	***P*-value**
Total	3,176	23	0.7	
Region				0.099
Eastern	1,269	5	0.4	
Central	976	7	0.7	
Western	931	11	1.2	
Age (years)				0.179
<60	2,206	13	0.6	
≥60	970	10	1.0	
Sex				0.468
Male	2,409	16	0.7	
Female	767	7	0.9	
Occupation				0.719
Civil servants	35	0	0.0	
Professional technicians	37	0	0.0	
Enterprise staff	101	0	0.0	
Self-employment	112	0	0.0	
Workers	424	3	0.7	
Farmers	1,331	9	0.7	
Unemployed or retired	186	2	1.1	
Students	78	2	2.6	
Others	872	7	0.8	
Cause of injury				0.235
Traffic accidents	962	9	0.9	
Sports	29	0	0.0	
Tumbles	865	3	0.3	
Falls from height	905	10	1.1	
Others	415	1	0.2	
Level of injury				0.159
Cervical	2,308	20	0.9	
Thoracolumbar	868	3	0.3	
Fracture or dislocations				0.387
No	1,153	6	0.5	
Yes	2,023	17	0.8	
Head injury				0.503
No	2,157	14	0.6	
Yes	1,019	9	0.9	
Chest injury				0.030
No	2,335	12	0.5	
Yes	841	11	1.3	
Abdominal visceral injury				0.038
No	3,072	20	0.7	
Yes	104	3	2.9	
Time before admission (hours)				0.862
<8	1,070	9	0.8	
8–24	709	5	0.7	
≥24	1,397	9	0.6	
ASIA score on admission				<0.001
A	634	15	2.4	
B	326	3	0.9	
C	923	2	0.2	
D	1,293	3	0.2	
Surgery				0.036
No	946	12	1.3	
Yes	2,230	11	0.5	

*ASIA, American spinal injury association*.

In patients who underwent surgery, there was no in-hospital death when surgery was performed within 12 h of the injury. However, no statistical difference was observed among groups with the following times before surgery: <12 h, 12–24 h, and ≥24 h (0/103, 0.0% vs. 5/732, 0.7% vs. 6/1,394, 0.4%, *P* = 0.719). Among patients who died in the hospital, most died from respiratory diseases (17/23, 73.9%), and six patients died from cardiovascular diseases (2/23, 8.7%), infections (2/23, 8.7%), and other reasons (2/23, 8.7%).

### Univariate and Multivariate Logistic Regression Analysis

Several factors were significantly associated with in-hospital mortality when we performed univariate logistic regression analysis, including chest injury (OR = 2.566, 95% CI: 1.128–5.837, *P* = 0.025), abdominal visceral injury (OR = 4.533, 95% CI: 1.325–15.501, *P* = 0.016), ASIA score on admission (OR = 0.404, 95% CI: 0.270–0.605, *P* < 0.001), and surgery (OR = 0.386, 95% CI: 0.170–0.878, *P* = 0.023). Age, sex, occupation, causes of injury, presence of fractures or dislocations, cervical spinal cord injury, head injury, and time before hospitalization and surgery were not associated with in-hospital mortality (Table 2).

**Table 2:** Univariate logistic regression analysis of clinical features and in-hospital mortality.

**Characteristic**	***P*-value**	**OR**	**95%CI**
Age	0.182	1.757	0.768–4.021
Sex	0.482	1.378	0.565–3.361
Occupation	0.458	1.044	0.931–1.171
Cause of injury	0.516	0.910	0.684–1.210
Fractures or dislocations	0.311	1.620	0.637–4.121
Cervical spinal cord injury	0.136	0.397	0.118–1.339
Head injury	0.469	1.364	0.588–3.162
Chest injury	0.025	2.566	1.128–5.837
Abdominal visceral injury	0.016	4.533	1.325–15.501
Time before hospitalization	0.572	0.874	0.549–1.393
ASIA score on admission	<0.001	0.404	0.270–0.605
Time before surgery	0.847	0.907	0.336–2.449
Surgery	0.023	0.386	0.170–0.878

*ASIA, American spinal injury association; OR, odds ratios; CI, confidence interval*.

Age, cervical spinal cord injury, chest injury, abdominal visceral injury, ASIA score on admission, and surgery were reanalyzed using multivariate logistic regression analysis. Cervical spinal cord injury (OR = 0.264, 95% CI: 0.076–0.917, *P* = 0.036), abdominal visceral injury (OR = 3.778, 95% CI: 1.038–13.755, *P* = 0.044), ASIA score on admission (A: reference; B:OR = 0.326, 95% CI: 0.093–1.146, *P* = 0.081; C:OR = 0.070, 95% CI: 0.016–0.308, *P* < 0.001; D:OR = 0.069, 95% CI: 0.019–0.246, *P* < 0.001), and surgery (OR = 0.341, 95% CI: 0.146–0.796, *P* = 0.013) were significantly associated with in-hospital mortality (Table 3).

**Table 3:** Multivariate logistic regression analysis.

**Characteristic**	***P*-value**	**OR**	**95%CI**
Cervical spinal cord injury			
Yes	NA	Reference	NA
No	0.036	0.264	0.076–0.917
Abdominal visceral injury			
No	NA	Reference	NA
Yes	0.044	3.778	1.038–13.755
ASIA score on admission			
A	NA	Reference	NA
B	0.081	0.326	0.093–1.146
C	<0.001	0.070	0.016–0.308
D	<0.001	0.069	0.019–0.246
Surgery			
No	NA	Reference	NA
Yes	0.013	0.341	0.146–0.796

*ASIA, American spinal injury association; OR, odds ratios; CI, confidence interval; NA, not available*.

### The Scale for Predicting the Risk of In-hospital Mortality

**Figure 1:**
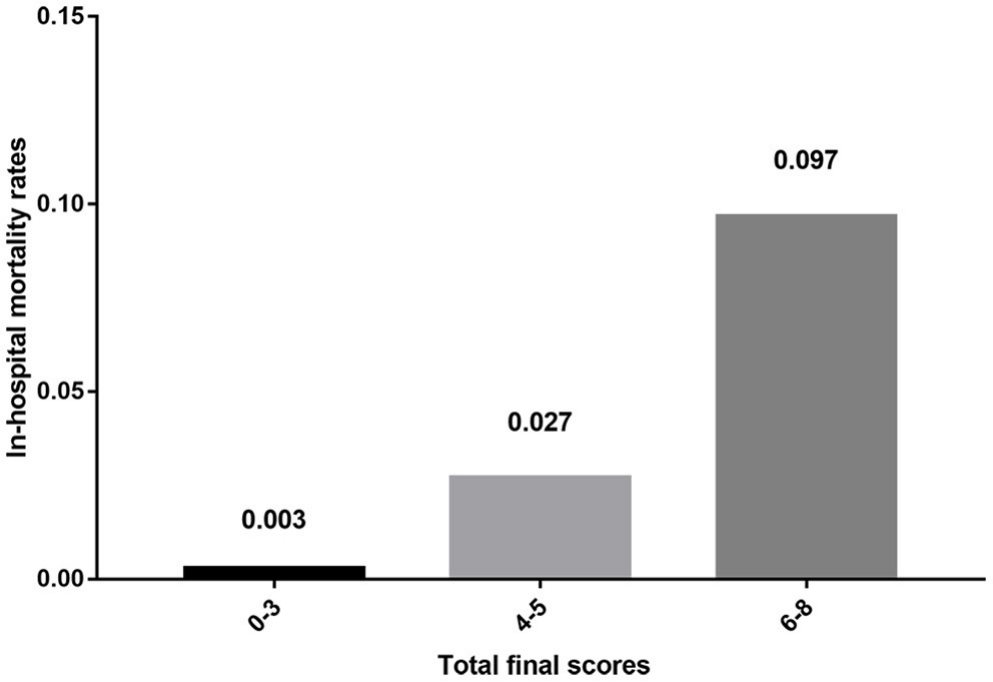
The in-hospital mortality risks of different groups.

The in-hospital mortality rates of patients with risk factors defined by multivariate logistic regression analysis were calculated, and primary scores for each of the four factors were derived (Table 4). The sum of the scores from all four factors was included in the scoring system and represented the risk of in-hospital mortality. The total scores ranged from 17 to 75 points; based on these scores, we divided patients into three groups (low-risk group: 17–39 points, moderate-risk group: 45–54 points, and high-risk group: 60–75 points). The in-hospital mortality risk rates of each group were 0.3% (low-risk group, 8/2,702, ranged from 0% to 1.887%), 2.7% (moderate-risk group, 12/443, ranged from 0% to 7.778%), and 9.7% (high-risk group, 3/31, ranged from 0% to 28.571%), respectively (*P* < 0.001) (Table 5). However, since the complicated scores were not convenient for clinical application, we simplified them mathematically and got final scores (Table 4). The resulting total scores ranged from 0 to 8 points, and the three ranges defining the aforementioned groups were 0–3 points (low-risk group), 4–5 points (moderate-risk group), and 6–8 points (high-risk group). The results of the in-hospital mortality risk in each group did not differ between the systems with primary scores and final scores (Table 5, Figure 1).

**Table 4:** The scale for predicting the risk of in-hospital mortality.

**Variables**	**In-hospital mortality rates (%)**	**Primary scores**	**Adjusted scores**	**Final scores**
Cervical spinal cord injury				
Yes	0.9	9	6	1
No	0.3	3	0	0
Abdominal visceral injury				
Yes	2.9	29	22	3
No	0.7	7	0	0
ASIA score of admission				
A	2.4	24	22	3
B	0.9	9	7	1
C	0.2	2	0	0
D	0.2	2	0	0
Surgery				
No	1.3	13	8	1
Yes	0.5	5	0	0

*ASIA, American spinal injury association*.

**Table 5:** The mortality rates of total primary and final scores.

**Groups**	**Cervical spinal cord injury**	**Abdominal visceral injury**	**Surgery**	**ASIA score of admission**	**Total primary scores**	**Total final scores**	**Mortality (%)**
Low-risk	–	–	+	C/D	17	0	0.257
	+	–	+	C/D	23	1	0.284
	–	–	+	B	24	1	0
	–	–	–	C/D	25	1	0
	+	–	+	B	30	2	1.149
	+	–	–	C/D	31	2	0.159
	–	–	–	B	32	2	0
	+	–	–	B	38	3	1.887
	–	–	+	A	39	3	0
	–	+	+	C/D	39	3	0
Moderate-risk	+	+	+	C/D	45	4	0
	+	–	+	A	45	4	1.167
	–	+	+	B	46	4	0
	–	–	–	A	47	4	4.878
	–	+	–	C/D	47	4	0
	+	+	+	B	52	5	0
	+	+	–	C/D	53	5	0
	+	–	–	A	53	5	7.778
	–	+	–	B	54	5	0
High-risk	+	+	–	B	60	6	0
	–	+	+	A	61	6	0
	+	+	+	A	67	7	28.571
	–	+	–	A	69	7	0
	+	+	–	A	75	8	16.667

*ASIA, American spinal injury association; “–”, without; “+”, with*.

## Discussion

In this study, we analyzed the in-hospital mortality among patients with TSCI and the associated factors and designed a new scale system for predicting the risk of in-hospital mortality using data from 59 tertiary hospitals in mainland China. In total, 3,176 patients were enrolled, and the in-hospital mortality rate of patients with TSCI and stable condition was 0.7% in mainland China. After performing multivariate logistic regression analysis, only cervical spinal cord injury, abdominal visceral injury, ASIA score on admission, and surgery were significantly associated with in-hospital mortality of TSCI. The new scale system contained the above four factors, and the in-hospital mortality risk of patients with 0–3 points (low-risk group), 4–5 points (moderate-risk group), and 6–8 points (high-risk group) was 0.3, 2.7, and 9.7%, respectively (*P* < 0.001).

Male patients were more commonly affected by TSCI, and the average male/female patient ratio was 3:1 in this study, which was similar to those found by other studies worldwide (21–23). This is probably because there are more males engaging in high-risk occupations than females (24). Further, elderly patients with degenerative changes in the cervical spine are vulnerable to slight injuries from falls or tumbles (25, 26). Therefore, special care should be provided to these patients. Regarding the level of injury in TSCI, both our study and previous ones showed that most patients had cervical spinal cord injury (26, 27).

In-Hospital mortality rates in TSCI have been reported to range from 1.2 to 40.5% and have been shown to be considerably higher in elderly patients than in younger patients (16, 17, 28–30). However, in this study, the in-hospital mortality rate of patients with TSCI was only 0.7%. This incidence of in-hospital mortality is surprisingly low when compared to published epidemiology studies of TSCI. We believe that this is partly due to discrepancies in the study population, inclusion criteria, and cultural differences. In mainland China, most patients were admitted from the emergency room for further treatment only after their condition stabilized. However, we only included inpatients, the emergency patients who were more likely to have a bad outcome were not included. This introduced a bias in the analysis of in-hospital mortality rate, which need to be cautious in further studies. Besides, surgery was performed more frequently in less severe patients. About 70.2% patients underwent surgery treatment in this study, which may be one of possible reasons for the lower in-hospital mortality rate. Further, we suppose it may be another possible reason, based on our anecdotal clinical observation, that patients who are dying or have no hope of treatment are more likely to leave the tertiary hospitals and return to local hospitals or go home to spend their last days. However, there is no literature evidence or exact data.

For inevitable TSCI, identifying the associated factors and administering effective treatments are crucial to decrease in-hospital mortality. In this study, we found that only cervical spinal cord injury, abdominal visceral injury, ASIA score on admission, and surgery were associated with in-hospital mortality. Several factors related to mortality have been reported, including older age, injury level, severity of neurological deficits, comorbidities, presence of other injuries, and ventilation status (14–17, 30). However, the severity of neurological impairment was the only reported common predictor among these studies. In this study, a less severe neurological impairment on admission (especially C and D) was associated with a decreased risk of in-hospital mortality. Early decompression and short transport time after TSCI were found to be associated with better neurological recovery in previous studies (31, 32). The role of surgical decompression after TSCI is to relieve the ongoing mechanical compression of the spinal cord, which can impair blood flow causing ischemia and neural tissue injury (5). We found that surgery was also beneficial in reducing the risk of in-hospital death. In the study by Inglis et al. (17), a lower in-hospital mortality rate was noted when surgery was performed (10% in the surgery group and 27% in the non-surgery group), and there was a marked difference in the time to death between patients who underwent surgery and those who did not (median time to death of 30 days in the surgery group and 7 days in the non-surgery group). However, surgery was performed more frequently in less severe patients, which may also contribute to the lower risk of in-hospital death in surgical patients. Besides, in this study, both cervical spinal cord injury and abdominal visceral injury were associated with an increased risk of in-hospital mortality.

This study had some limitations. As we discussed above, we only included hospitalized patients with stable condition, so the corresponding mortality and risk factors are more appropriate for these patients. But put it another way, the in-hospital mortality rate for these stabilized patients contributed to literatures. Since this study is a secondary data analysis of previous national epidemiological investigation of TSCI, more specific information, such as Glasgow coma scales for patients with head injury, were not included in the analysis. Although, compared with previous studies, a large sample size was acquired, a small proportion of patients experienced in-hospital mortality. Hence, this study does not have sufficient power to make inferences on several factors. Besides, surgery tended to be performed in less severe patients, disturbing the conclusion that surgery reduced the risk of in-hospital mortality. Since there have been other studies demonstrating the neurological and survival benefits of surgery, we recommend performing surgery in stable patients with TSCI when feasible. For the new scale system, it may only be suitable for evaluating patients with stable condition, and its reliability needs to be further verified by other studies. Due to the small proportion of patients experienced in-hospital mortality, there is some bias in mortality rate for each situation. When divided into several different situations, there is no mortality for some situations, even in the high-risk group.

In conclusion, the in-hospital mortality rate of patients with TSCI and stable condition in mainland China was shown to be 0.7%. Cervical spinal cord injury, abdominal visceral injury, ASIA score on admission, and surgery were significantly associated with in-hospital mortality in these patients. The proposed scale system may be beneficial for clinical decision-making and for communicating relevant information to patients and their families.

## Data Availability Statement

The raw data supporting the conclusions of this article will be made available by the authors, without undue reservation.

## Ethics Statement

The studies involving human participants were reviewed and approved by Ethics Committee of Honghui Hospital. Written informed consent from the participants' legal guardian/next of kin was not required to participate in this study in accordance with the national legislation and the institutional requirements.

## Author Contributions

YG: conceptualization, visualization, formal analysis, methodology, software, and writing—original draft. JD: investigation and project administration. DH: funding acquisition, project administration, resources, supervision, and validation. BH: funding acquisition, resources, supervision, and validation. YC and BZ: data curation and analysis. XG: data curation and visualization. LY: conceptualization, funding acquisition, investigation, project administration, resources, supervision, validation, review, and editing. All authors contributed to the article and approved the submitted version.

## Funding

This study was supported by the National Natural Science Foundation of China (Grant No. 81830077).

## Conflict of Interest

The authors declare that the research was conducted in the absence of any commercial or financial relationships that could be construed as a potential conflict of interest.

## Publisher's Note

All claims expressed in this article are solely those of the authors and do not necessarily represent those of their affiliated organizations, or those of the publisher, the editors and the reviewers. Any product that may be evaluated in this article, or claim that may be made by its manufacturer, is not guaranteed or endorsed by the publisher.
