# Supplementary material for: A New Scale for Predicting the Risk of In-hospital Mortality in Patients With Traumatic Spinal Cord Injury
Source: Front Neurol. 2022 Jun 2;13:894273. doi: 10.3389/fneur.2022.894273 (PMC9204840; doi:10.3389/fneur.2022.894273)
Supplement: Supplementary file 1 [file Data_Sheet_1.PDF]

List of ICD-10-CM Codes for Traumatic Spinal Cord Injury (TSCI) Diagnosis

**ICD-10-CM Code**	**ICD-10-CM Definition**
S12.001	First cervical fracture (atlas)
S12.101	Second cervical fracture (closed)
S12.111	Second cervical fracture (open)
S12.701	Multiple cervical fracture with spinal cord injury
S12.702	Multiple fractures of cervical vertebra
S12.901	Cervical vertebral fracture
S13.101	Cervical dislocation
S13.102	Atlantoaxial subluxation
S14.101	Cervical spinal cord injury
S22.001	Thoracic vertebra fracture
S22.011	Thoracic vertebra fracture (open)
S22.101	Thoracic multiple fracture
S23.101	Thoracic vertebra dislocation
S24.101	Thoracic spinal cord injury
S32.001	Lumbar vertebrae fracture
S32.701	Multiple fractures of lumbar vertebrae and pelvis
S33.101	Lumbar dislocation
S34.101	Lumbar spinal cord injury
S34.301	Cauda equina injury
S34.401	Sacral spinal cord injury
S32.101	Sacral fracture
G37.302	Transverse injury of spinal cord
T09.302	Spinal cord injury

Note: Injury to nerve roots codes not included
